# New Appliances and Things Medical

**Published:** 1896-08-29

**Authors:** 


### NEW APPLIANCES AND THINGS MEDICAL.

[We shall be glad to receive, at our Office, 428, Strand, London, W.C., from the manufacturers, specimens of all new preparations and appliances, which may be brought out from time to time.]

### HEALTH NIGHT-LIGHTS.

(Fowler's Patent Night-light Company, Planet Works, London, S.E.)

This is a new departure in the manufacture of night-lights. Incorporated with the paraffin wax of which they are composed is a dibromo-compound, which is decomposed in the flame, liberating free bromine. The object of the free bromine is that it should become diffused in the air of the room, operating at once as an antiseptic and as stimulant to respiration. From practical tests to which we have submitted these night-lights we find that a small quantity of bromine is liberated, as may be at once detected by the characteristic odour. Although the total amount thus set at liberty during the period of combustion is comparatively small, it probably exercises a certain influence on the atmosphere into which it passes by oxidising organic impurities and thus discouraging bacterial growth and activity. Under ordinary conditions of the lungs, it causes a gentle stimulation of the mucous membranes of the respiratory passages, which, under certain conditions may conceivably be of advantage to the patient, although there are irritable conditions of the mucous membranes which could not tolerate the presence of even such small quantities of bromine as become diffused into the air during the eight hours of combustion. With this reservation, we are prepared to recommend these night-lights for general and sick-room purposes.

### ANTISEPTIC ACCOUCHEMENT SETS.

(S. Maw, Son, and Thompson, 7 to 12, Aldersgate Street, E.C.)

We have examined a complete set of the above, and regard the selection of articles as well chosen and sufficient for the purpose. The antiseptic towels with waistband, as well as the other varieties of towels and napkins are all first-rate, with good absorbent qualities, and likely to be of great service both to the nurse and doctor in carrying out their duties in accordance with the strictest antisepsis. As may be seen from the list of contents, the supply of towels and napkins is sufficient, if not liberal; the mackintosh sheets, both for floor and bed, are excellent of their kind, and the antiseptic accouchement sheets are respectively 42 inches by 36 inches and 30 inches by 27 inches, and in other respects just what the most experienced nurses fabricate for themselves at considerable trouble and expense. With regard to the other accessories and et ceteras, they are entirely adequate and satisfactory. At our suggestion, Messrs. Maw are providing two antiseptic binders and perchloride in a more soluble form. We consider this new departure on the part of these well-known manufacturers will be received with gratitude by doctors, nurses, and patients alike, since by the outlay of 25s., without the fear of any necessary article being omitted, a complete set, and of the best quality, can be had at a moment's notice.

